# Toward Homogenous Antibody Drug Conjugates Using Enzyme-Based Conjugation Approaches

**DOI:** 10.3390/ph14040343

**Published:** 2021-04-08

**Authors:** Ahmad Fawzi Hussain, Armin Grimm, Wenjie Sheng, Chaoyu Zhang, Marwah Al-Rawe, Karen Bräutigam, Mobarak Abu Mraheil, Felix Zeppernick, Ivo Meinhold-Heerlein

**Affiliations:** 1 Department of Gynecology and Obstetrics, Medical Faculty, Justus-Liebig-University Giessen, 35392 Giessen, Germany; armin.Grimm@med.uni-giessen.de (A.G.); Wenjie.Sheng@med.uni-giessen.de (W.S.); Chaoyu.Zhang@med.uni-giessen.de (C.Z.); marwah.al-rawe@gyn.med.uni-giessen.de (M.A.-R.); felix.zeppernick@gyn.med.uni-giessen.de (F.Z.); Ivo.Meinhold-Heerlein@gyn.med.uni-giessen.de (I.M.-H.); 2 Department of Gynecology and Obstetrics, University Medical Center Schleswig-Holstein, Campus Lübeck, 23562 Lübeck, Germany; karen.braeutigam@uksh.de; 3 Institute of Medical Microbiology, German Center for Infection Research, Partner site Giessen-Marburg-Langen, Justus-Liebig University Giessen, 35392 Giessen, Germany; Mobarak.Mraheil@mikrobio.med.uni-giessen.de

**Keywords:** antibody, antibody drug conjugate, enzyme-based conjugation

## Abstract

In the last few decades, antibody-based diagnostic and therapeutic applications have been well established in medicine and have revolutionized cancer managements by improving tumor detection and treatment. Antibodies are unique medical elements due to their powerful properties of being able to recognize specific antigens and their therapeutic mechanisms such as blocking specific pathways, antibody-dependent cellular cytotoxicity, and complement-dependent cytotoxicity. Furthermore, modification techniques have paved the way for improving antibody properties and to develop new classes of antibody-conjugate-based diagnostic and therapeutic agents. These techniques allow arming antibodies with various effector molecules. However, these techniques are utilizing the most frequently used amino acid residues for bioconjugation, such as cysteine and lysine. These bioconjugation approaches generate heterogeneous products with different functional and safety profiles. This is mainly due to the abundance of lysine and cysteine side chains. To overcome these limitations, different site-direct conjugation methods have been applied to arm the antibodies with therapeutic or diagnostics molecules to generate unified antibody conjugates with tailored properties. This review summarizes some of the enzyme-based site-specific conjugation approaches.

## 1. Introduction

Antibodies are produced by B-lymphocytes and are part of the adaptive immune system [1]. They occur for example in the blood circulation and the mucosa, and they have the ability to recognize and bind potential harmful specimens by specific molecular moieties, called antigens. Antibodies can be divided into five antibody-isotypes: IgG, IgA, IgM, IgE, and IgD, which perform specific functions in the humoral immune system [2]. 

**Figure 1:**
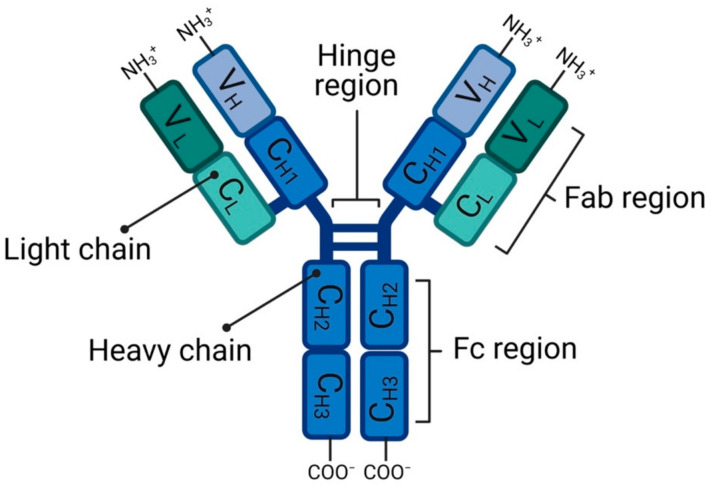
Schemes structures of monoclonal antibody (IgG isotypes) [3,4]. The figure was created with BioRender.com (accessed on 26 February 2021).

The structural prototype of all antibody isotypes is nearly identical and corresponds to the structure of IgG antibodies. Furthermore, IgG is the most commonly used isotype for antibody engineering, which we will focus on in the following part with respect to its structure. IgG molecules consist of two heavy chains and two light chains, which are composed of different variable and constant immunoglobulin domains. Each domain is composed of a beta-sheet structure and is stabilized through intramolecular disulfide-bridges. Each heavy chain is made up of one variable heavy (VH) domain and three constant heavy (CH1, CH2, CH3) domains, while the light chain has one variable light domain (VL) and constant light domain (CL) (Figure 1) [3]. 

Based on the enzymatic cleavage by the pepsin enzyme, the antibody structure can be divided into two functionally different regions. One fragment crystallizable (Fc) region and two fragment antigen binding (Fab) regions. Both parts are connected with each other by a hinge-region between the CH1- and CH2-domain (Figure 1) [4]. The Fab-fragment is built by the light chain and the VH and CH1 of the heavy chain. They are connected to each other through a disulfide bridge between the CH1 and the CL domain. The N-terminal ending of the Fab-fragment is also referred to as fragment variable (Fv). It contains the domains, which are relevant for the antigen binding. The variable domains of the heavy and the light chain have specific regions, which vary significantly in their amino acid sequence. These hypervariable regions compose the binding site of the antibody to its antigen and are called the complementary-determining-regions (CDR). Each variable domain incorporates three CDR. The structure of the CDR is stabilized by the other domains of the variable region [4].

The Fc-fragment is solely built by the heavy chain of the antibody and consists of two immune globulin domains (CH2 and CH3). Additionally, the Fc-fragment shows at least two branched side chains, each containing about nine hexose molecules. The asparagine at position 297 (sp297) of the Fc-fragment is glycosylated. The Fc-fragment interacts with the Fc-receptor on the surface of various immune cells like macrophages and is crucial for the mediation of the effector function of the antibody. Depending on the corresponding immune cell, there are several different effector functions reaching from an improved opsonization of the bound antigen to the activation of antibody-dependent cellular cytotoxicity [5,6].

In the last decades, antibody-based diagnostic or therapeutic applications are well established in medicine due to their powerful properties to recognize specific antigens and their therapeutic mechanisms such as blocking specific pathways, antibody-dependent cellular cytotoxicity, and complement-dependent cytotoxicity [7]. 

The recent development in genetic modification techniques have paved the way for improving antibody properties and to develop new classes of antibody-based diagnostic and therapeutic agents [8]. These techniques allow arming antibodies with various effector molecules such as fluorescent proteins, toxic proteins, fluorophores, nanoparticles, radio agents, and chemotherapeutic agents. 

Recently, an antibody isotype swapping approach emerged as an elegant solution to reduce adverse effects and improve antibody accumulation into targeted tissues. Previous studies indicate that this approach warrants further studies to test its efficacy in preclinical in vivo applications [9,10]. Furthermore, different strategies have been developed to increase antibody half-life circulation stability, such as increasing its affinity to FcRn [11,12].

Antibody drug conjugates (ADCs) are the best-established class of antibody conjugates; therefore, we will discuss it in this review as an example for antibody conjugate. 

## 2. ADCs 

ADCs represent one of the most promising strategies to selectively eliminate cancer cells by combining the specific cell receptor targeting of a monoclonal antibody (mAb), with a highly potent cytotoxic agent.

The origin of ADCs can be traced back to 1913 to the German scientist Paul Ehrlich, who coined the term “magic bullet” and proposed the concept of tumor targeting therapy. Approximately 50 years later, the magic bullet concept was realized by conjugating methotrexate to an antibody targeting leukemia cells. This and other constructs showed only limited success due to their higher immunogenicity, low therapeutic activity, low specificity, and stability [13].

Currently there are three ADCs that have been approved by the FDA for a therapeutic use. The first FDA-approved ADC is Trastuzumab emtansine (TDM-1; Kadcyla), which is based on conjugating the humanized anti-human epidermal growth factor receptor 2 (Her2)-IgG1 antibody trastuzumab with the maytansinoid, also called DM1 using a stable non-cleavable linker. Maytansinoid is a microtubule-targeted drug that binds to tubulin and depolymerize microtubules and arrests cells in mitosis [14]. This ADC is used as a second-line therapy in Her2-positive patients, which have previously been treated with trastuzumab and taxane-based chemotherapy [15]. The Second FDA-approved ADC is Brentuximab vedotin (Adcetris), which is an anti-CD30 monoclonal antibody (mAb) conjugated to Monomethyl auristatin E (MMAE), an antimitotic agent which inhibits cell division by blocking the polymerization of tubulin via a cleavable peptide linker. It is licensed for the therapy of relapsed or refractory Hodgkin lymphoma after autologous stem cell transplant or if autologous stem cell transplant is contraindicated [16]. 

The third ADC approved for clinical use is Inotuzumab ozogamicin (CMC-544; Besponsa), which is composed of humanized monoclonal IgG4 antibody targeting CD22 equipped with cytotoxic antibiotic N-acetyl-gamma-calicheamicin dimethylhydrazine via 4-(4′-acetylphenoxy)butanoic acid (AcBut) cleavable linker. CMC-544 is currently used as a monotherapy to treat adult patients with relapsed/refractory B-cell acute lymphoblastic leukemia [17,18].

To generate an antibody drug conjugates five elements need to be taken into consideration, which will now be discussed. 

### 2.1. Antibody 

The used antibody should have a high specificity and affinity (Kd < 10 nM) to the target antigen. Furthermore, there should not be any cross reactivity with other antigens or unspecific antigen binding. These ensure a high selectivity of the antibody for the targeted tissue controlling the systemic effects of the antibody and its interaction with healthy tissues. An unspecific binding of the antibody to various antigens could increase the incidence of side effects and decrease the intended therapeutic or diagnostic effect due to a reduced amount of ADC that would reach the targeted tissue, leading to a decreased therapeutic index. It is also important to investigate the immunogenic potential of the used antibody. Generally, it can be assumed that human or humanized antibodies will show a lower immunogenicity than chimeric or murine antibodies [19]. Even after conjugation with a drug, antibodies can retain their original activity like antibody-dependent cellular cytotoxicity. Those effects can be complementary or oppositional to the wanted effect. Therefore, the independent activity of the antibody should be investigated and considered before conjugation [16]. 

### 2.2. Target Antigen 

There are different strategies that can be followed to bring the ADC to the target tissue. The most common approach is to target an antigen on the surface of tumor cells. The benefit here is that the tumor is directly affected by the cytotoxicity of the payload. An important disadvantage is that the malignant cells can decrease the efficacy of the ADC by resistance mechanisms, such as receptor downregulation or multidrug resistance. To address these challenges, the targeting of the tumor stroma and vasculature can be a useful alternative leading to indirect damage of the tumor. Considering that a tumor consists of heterogeneous populations, ADCs can also be designed to selectively attack specific subpopulations like aggressive cancer stem cells (CSC), which are suspected of being responsible for tumor growth, metastasis, and recurrence. 

Generally, a high specificity of the chosen antigen for the targeted tissue is characterized by a high expression in the targeted tissue, while only showing a low expression in healthy tissue. Otherwise, it would not be possible to achieve a good discrimination between healthy and pathological tissues. In addition, the selected antigen must be internalized to the cell cytoplasm. Therefore, this antigen is approachable for ADCs circulating in the blood system and after binding the ADC-antigen-complex is internalized. Here, it must be considered that the internalization and degradation behavior of the ADC-antigen-complex can differ depending on the exact epitope targeted on the antigen. Antigen shedding should also be avoided, as it would induce antigen binding during circulation. Also, it should be known whether the antigen expression is homogeneous or heterogeneous in the tumor tissue. Achieving a good therapeutic efficacy when facing a heterogeneous expression can be accomplished by inducing a higher bystander effect, but this could also increase unwanted systemic side effects [16,19].

### 2.3. Linker 

The most important feature for the linker is the stability in the blood plasma to avoid a systemic release of the payload, which would cause more side effects as well as reduce the efficacy and the therapeutic index of the ADC. Linkers can be categorized into cleavable and non-cleavable linkers. Cleavable linkers can for example be split in the acidic environment of the lysosomes and therefore ensure a controlled releasing of the payload from the antibody. Aiming a higher bystander effect, the use of cleavable linkers is often favorable due to the ability to retain membrane-permeable properties of the payload. ADCs with non-cleavable linkers must undergo a complete proteasomal degradation before the payload is released. In some cases, this degradation does not lead to a complete separation of the payload from the antibody, so residues of the antibody can remain attached to the drug molecule. The remaining antibody residues could potentially interact with the function of the drug [16,19]. 

### 2.4. Drug 

The considered drug must be suitable for the conjugation to an antibody, therefore it should either already have an appropriate conjugation side or it should be accessible for an adequate modification that does not affectthe function of the drug [19]. The most commonly used drugs for therapeutic approaches are microtubule inhibitors like auristatins and DNA damaging drugs such as Maytansinoid and MMAE. Also, radionuclides can be functionalized as cytotoxic payloads [16]. An important impact on the usability of ADCs arises from the drug-to-antibody ratio (DAR). Even though the optimal DAR can differ depending on the other properties of the ADC, most approaches aim for a DAR of 2–4 [20]. This is supported by the finding, that a high DAR causes instable ADCs with increased plasma clearance, reduced half-life and more systemic side effects [16]. The amount of ADC that reaches the targeted tissue can be relatively low compared to the injected dose. Due to this, the chosen drug should have a high potency to ensure the wanted effect in the target even at a low concentration. Therefore, it is necessary to estimate the expected concentration in the targeted tissue [19].

### 2.5. Conjugation Method

Random conjugation methods are usually based on direct functionalization of lysine or cysteine residues of the antibody for conjugation. Although these methods are easy to use, they yield heterogeneous mixtures of ADCs with different pharmacological properties. Lysine residues are found about 40 times per antibody. Therefore, over 10^6 different ADC species can be produced. Even though cysteine residues occur eight times per antibody on average, this approach also leads to potentially over 100 different ADCs [16]. Hence, it can be difficult to estimate the generated ADC pharmacokinetics or an unwanted systemic release of the drug [21,22]. One promising approach to solve this issue is the use of site-specific conjugation methods, which lead to homogeneous products with a defined DAR. 

In the last decades, several site-specific conjugation methods have been developed. They are aiming to equip the antibody with a unique reaction site, which can then be used for the conjugation. The used methods can be generally categorized in the following way: cysteine-conjugation, glycoconjugation, incorporation of unnatural or non-canonical amino acids, and enzyme-based conjugation methods [21]. 

Cysteine-conjugation methods are based on the usage of reduced solvent accessible cysteine residues and their conjugation with electrophiles like maleimides or haloacetamides. To achieve this, several methods have been developed. The reduction/alkylation procedure for example tries to overcome the limitations of primary cysteine conjugation methods by functionalizing all eight interchain cysteines. For this purpose, it is necessary to reduce the interchain disulfide bridges in a first step. The resulting free cysteines could then be used for the conjugation. A main limitation of this procedure is that it leads to ADCs with a DAR of 8, which showed a decreased maximal tolerable dose and circulation half-life compared to ADCs with a DAR of 4 and equivalent activity [20].

Another promising approach based on cysteine conjugation is the insertion of surface accessible cysteine residues. For this purpose, Junutula et al. developed a phage display (PHESELETOR) that allows the identification of engineered cysteine residues that are free for conjugation while simultaneously not interfering with the antibody structure or antigen binding [23]. Their new conjugation method also overcame the blocking of engineered cysteine residues by cysteinylation or glutathionylation. A partial reduction of the modified antibodies followed by the reoxidation of interchain disulfide bonds in the presence of CuSO4 produced free engineered cysteines which could then be used for conjugation. These THIOMABs lead to homogeneous ADCs with a DAR of 2.0 and showed equivalent efficacy as ADCs with a higher DAR produced by conventional thiol conjugation. Additionally, THIOMABs achieved a better therapeutic index in animal experiments and showed improved serum stability than conventional ADCs [21,24].

Glycan conjugation and glycoengineering approaches are based on the fact that human IgG has a conserved glycosylation site at position N297. This site is not only usable for site-specific conjugation. Due to its distance to the antigen binding site, it ensures that the antigen binding will not be affected by the conjugation. A main challenge in this approach is to generate homogeneous glycans, given that glycosylation is a heterogeneous posttranslational modification. An example for glycan-based method is the periodate oxidation of glycans to produce aldehydes. These aldehydes can be used for conjugating small molecule with hydrazide and aminooxy compounds [21]. Li et al. followed another approach by modifying sialic acid with a bioorthogonal functional group and subsequently incorporated it in an antibody by the enzyme sialyltransferase. This method achieved a DAR of 4–4.5 [25]. A disadvantage of glycoconjugation approaches is that they have no flexibility in the number or localization of the modification. Also, it has to be considered that monosaccharides like the sialic acid can cause a higher immunogenicity [21].

Unnatural or non-canonical amino acids are inserted into the antibody by ribosomal incorporation. To do this, a mutated protein is encoded of which DNA sequence bears the amber stop codon (TAG). The TAG codon is inserted at the localization where the unnatural amino acid should be built in. The expression of this protein is done in the presence of a special tRNA/aminoacyl-tRNA-synthetase pair which can insert the unnatural amino acid at the amber stop codon site [21]. These and other site-specific conjugation methods were reviewed elsewhere [26]. In this review we will summarize some of the enzyme-based conjugation approaches. 

## 3. Enzyme-Based Conjugation Approaches

Controlled enzymatic conjugation methods such as sortase, transglutaminase, formylglycine-generating enzyme and O6-Alkylguanine-DNA alkyltransferase (AGT, also called SNAP-tag method) have emerged as promising alternative site-specific conjugation methods.

These conjugation methods offer a nearly defined DAR and site directed conjugation of the effector molecules, which are associated with a unified pharmacokinetic, safety, and efficacy properties of the generated antibody conjugates. Below is a short description of some of the enzyme-based conjugation methods. 

### 3.1. Sortases

**Figure 2:**
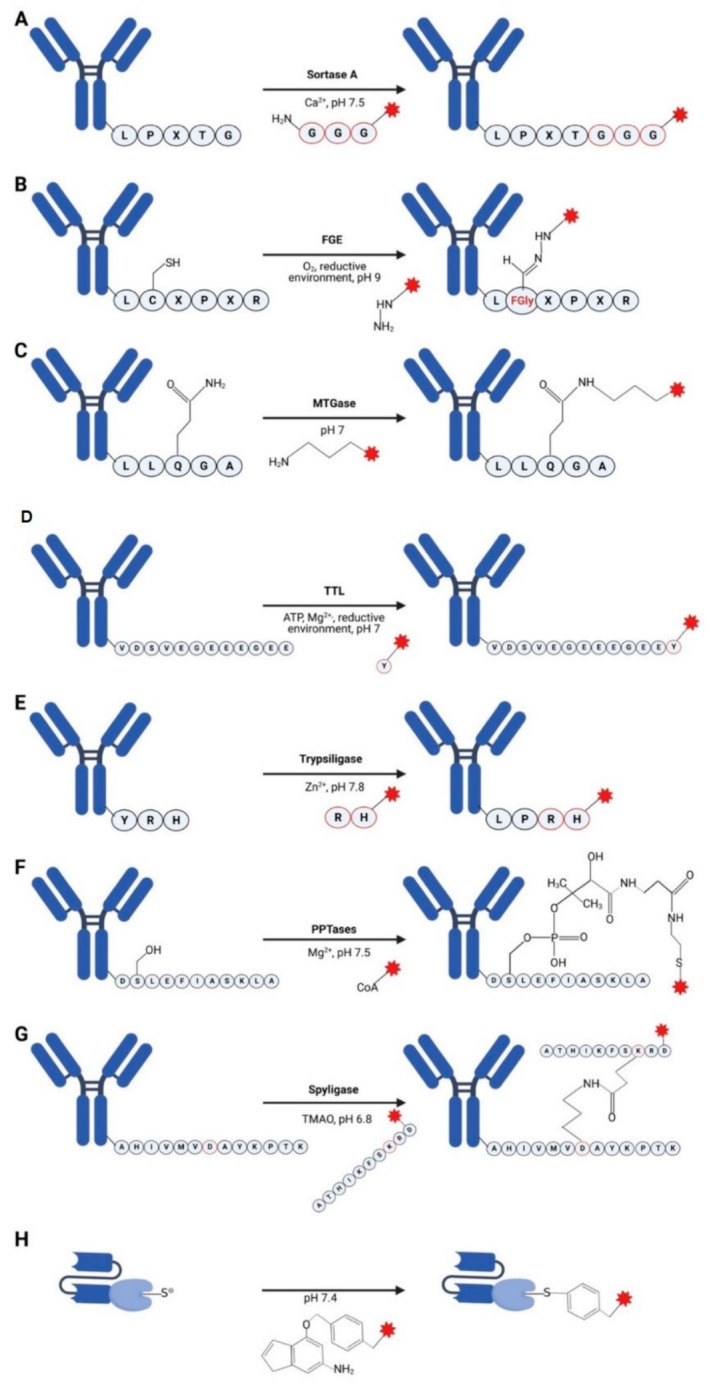
Different enzyme-based site-specific conjugation methods. (**A**) Antibody conjugation using sortase A [32]. (**B**) Scheme for the attachment of effector molecule to antibody using formylglycine-generating enzyme (FGE) [34,35]. (**C**) Modifying antibody using microbial transglutaminase (MTGase) [36]. (**D**) Generating antibody drug conjugates ADC using tubulin tyrosine ligase (TTL) [37]. (**E**) Allying trypsiligase for conjugating effector molecules to antibody [38,39]. (**F**) Antibody conjugation using phosphopantetheinyl transferases (PPTases) [40,41,42]. (**G**) Antibody modification with SpyLigase [43,44]. (**H**) SNAP-tag mediated conjugation of antibody-SNAP fusion proteins with BG-modified effector molecule [45,46,47]. The figure was created with BioRender.com (accessed on 26 February 2021).

Sortase is a special transpeptidase that can link to the Gram-positive bacteria cell wall via anchoring to surface proteins. Most sortases can be classified into six distinct subfamilies, called class A–F enzymes [27,28]. Sortase A (SrtA) plays an important role in different stages of the pathogenic process in many Gram-positive bacterial infection [28]. As an extracellular membrane enzyme, SrtA can anchor a surface protein which typically includes two topogenic sequences, N-terminal signal peptides and C-terminal sorting signals. C-terminal modified with sequence LPXTG (with X being any amino acid) can be recognized by Srt A, which leads to the amide bond cleavage between the threonine (T) and the glycine (G), followed by the nucleophilic attack of oligoglycine substrates and the formation of a thioester acyl-enzyme intermediate (LPXT–SrtA) (Figure 2A). Therefore, the surface protein is finally anchored to the cell wall peptidoglycan which is then incorporated into the envelope and exposed on the microbial surface [29]. Mao et al. originally showed that the sortase-catalyzed transpeptidation made a contribution to protein ligation using recombinant green fluorescence protein (GFP) with a C-terminal LPETG-His6 tag as a model protein. The specific conjugation with peptides by a SrtA-catalyzed transpeptidation was then revealed [30]. Hidde Ploegh’s group explained a concept of sortagging or sortase-mediated ligation (SML), which use sortase-mediated transpeptidation to label proteins with a variety of molecular probes in a site-specific way [31]. Thus, engineering SrtA is ideal and potential in antibody drug conjugation. Pan and his colleagues conjugated a triple glycine-modified monomethyl auristatin E (MMAE) to anti-CD20 ofatumumab (OFA) equipped with a short C-terminal LPETG tag at heavy chain (HL) via SrtA-mediated transpeptidation. The generated ADCs showed extremely strong potency in killing CD20 positive cancer cells, even though CD20, a non-internalizing epitope, was not considered as an ideal target for ADCs. However, the major disadvantage of SrtA-mediated transpeptidation is the low catalytic efficiency, which needs a huge excess of nucleophilic oligoglycine-modified toxins (100-fold molar excess) and enzyme to generate ADCs since the reaction is reversible [32]. Later, the same research group developed a chemo-enzymatic approach, incorporating the sortase mediated ligation and the strain promoted azide-alkyne cycloaddition (SPAAC). First, LPETG-tagged antibody was equipped with a bifunctional oligoglycine-modified small molecule by SrtA-mediated ligation. The toxin was modified with a desired functional group and conjugated to the antibody by SPAAC in the second step. The homogeneous ADC produced by this two-step approach could be internalized into CD20+ tumor cells effectively. The results also demonstrated that the efficacy of such chemo-enzymatic conjugates was superior to those prepared via a direct enzymatic coupling method due to the higher DAR. In addition, this two-step procedure exhibited a potency to be readily scaled up on account of cost reduction and less toxic waste, providing a new strategy to find various applications from protein modifications to functionalized materials for ADCs [33]. 

### 3.2. Formylglycine-Based Methods

Formylglycine-generating enzyme (FGE) can bind to a sequence (CXPXR, X is usually serine, threonine, alanine, or glycine) and catalyze the oxidation of cysteine to a formylglycine (fGly), which is a post-translational modification (PTM) derived from catalytic cofactors [48]. This non-canonical residue can be generated within any desired target protein and subsequently can be used for bioconjugation chemistry, especially for preparation of ADCs (Figure 2B). Carrico IS et al. engineered a six amino acid tag (LCTPSR) as an “aldehyde tag,” which can be reversibly modified with multiple epitopes [49]. The aldehyde tag was further developed in 2014. Drake and his colleagues demonstrated the use of Hydrazino-iso-Pictet-Spengler (HIPS) chemistry to conjugate maytansine payloads to aldehyde tags which had been genetically inserted into trastuzumab. The HIPS can produce a stable, covalent C−C bond between the cytotoxin payload and the antibody. This C–C bond in ADCs is supposed to be stable when encountered with proteases, low pH, and reducing reagents under circulation and neonatal FC receptor (FcRn) recycling. Also, the HIPS chemistry is less affected at different conjugation sites [34]. However, other portions of the linker may influence stability and efficacy of ADCs. Another strategy uses pyrazolone derivatives that can also strengthen the C–C bond forming conjugation exists in Knoevenagel-type reactions with aldehyde-bearing proteins. There are two subtypes for Knoevenagel-type reactions. The first subtype is trapped-Knoevenagel ligation, which is trapped by intramolecular attack of a thiol. The second subtype is tandem-Knoevenagel ligation which is based on adding a second equivalent of a pyrazolone nucleophile [50]. As the tandem-Knoevenagel ligation can generate ADCs with higher DAR, Kudirka R et al. presented a chemical ligation that involved the double addition of a pyrazolone moiety with an aldehyde-labeled protein, Knoevenagel condensation–Michael addition (TKM), and generating a new structure without a catalyst. They generated three TKM ADCs with a DAR of 4, which showed efficient internalization and release of the cleavable payload [50]. This study showed that a ligation chemistry which afforded a prepared bivalent molecule would improve ADC generation and the bioconjugation chemistry. Krüger et al. showed a new aldehyde tag technology to attach different payloads. This method allowed two different recognition sequences selectively addressed by different formylglycine generating enzymes. An iron sulfur protein AtsB which forms Methanosarcina mazei (MM-AtsB) can recognize sequence CTAGR with an alanine instead of a proline, and a sequence CTPSR for classical FGE is utilized as well. These two different enzymes enable generation of two-fold modification of target proteins and successively introduce two distinct payloads to a target protein. Meanwhile, the target protein retains its function even with the loaded tag and the enzymatic transformation [35]. 

### 3.3. Transglutaminases

Transglutaminases are a class of enzymes that can be found both in bacteria and humans. Microbial transglutaminase (MTGase), from Streptomyces mobaraensis, is probably the most employed transglutaminase for biotechnology, due to its broader substrate specificity, lower deamidation activity, wider range of reaction pH, and temperature. The ability of MTGase to catalyze the formation of amide bonds between a γ-carboxyamide group of a glutamine and a primary amine has now been used to produce homogeneous ADC (Figure 2C) [51].

Dennler et al. utilized a two-step strategy to synthesize homogeneous trastuzumab-MMAE with the DAR of 2. First, a glycan in asparagine (N297) beside glutamine 295 (Q295) within the heavy chain was removed, which made the conjugation site (Q295) specifically recognized by MTGase, and a linker containing azide function was connected to an antibody. Then, MMAE was attached to the modified antibody through SPAAC. Compared to thiol-maleimide approach, ADCs generated by an SPAAC approach do not need to be deacetylated and are more stable in vivo. The research group also confirmed that the trastuzumab-MMAE could specifically bind to HER2+ cells (BT-474, SK-BR-3) and efficiently kill tumor cells with an IC50 of 89.0 pM and 21.7 pM, respectively [36]. 

Nevertheless, deglycosylation may reduce stability of antibodies, resulting in increased antibody aggregation [52]. Recently, Ebenig et al. coupled a novel recognition tag SPI7G derived from a native MTGase substrate Streptomyces papain inhibitor (SPIP), in which the Lys7 was substituted by glycine to trastuzumab and further armed with MMAE by SPAAC. The group screened seven oligopeptides (wtSPI, SPI7G, SPI7R, GEN, GEC, 9mer, and LLQG), and proved SPI7G had the highest turnover rate, as well as superior labeling after being coupled to trastuzumab. The ADC assembled from trastuzumab- SPI7G showed fourfold increased DAR of 1.81 compared to trastuzumab-LLQG (0.48). Furthermore, the modified trastuzumab-MMAE obtained the ability to kill SK-BR-3 breast cancer cells efficiently with IC50 value of 151 pM, which is 12-fold higher than that of trastuzumab-LLQG [53]. 

As the ADCs combined the technology of chemotherapy and monoclonal antibody, its therapeutic activity could be affected by cell drug resistance. Walker et al. synthesized bifunctional antibody conjugates through SPAAC and IEDDA in one-pot synthesis, in which trastuzumab is modified by pharmaceutically relevant polyethylene glycol (PEG) polymer and cytotoxic payload DM1. The dual modified trastuzumab was confirmed to have no negative impact on the toxicity in SKOV3 cells, since the IC50 value of dual labeled conjugate was about 6.6 nM, compared to 8.1 nM of the DM1-loaded conjugate [54]. This research revealed the potency of antibody conjugates containing varied functionalities. Despite these merits, the pharmacokinetics, conjugate stability in vivo, and drug safety of these multifunctional antibody conjugates should be taken into consideration. 

### 3.4. Tubulin Tyrosine Ligase

Tubulin tyrosine ligase (TTL) is an enzyme that mediates a novel site-specific protein functionalization termed Tub-tag labeling, recognizing a 14-amino-acid hydrophilic tag (VDSVEGEGEEEGEE, Tub-tag) at the C-terminus of a protein of interest (POI) and catalyzing the addition of tyrosine derivative [55]. The tyrosine derivative contains a unique chemical entity that can be functionalized through chemo-selective conjugation such as SPAAC (Figure 2D) [37]. Schumacher et al. established a modular and high-yielding TTL based site-specific modification method by fusing Tub-tag sequence to GFP-specific nanobodies (GBP4 and GBP1) and GFP. They then successfully ligated unnatural tyrosine derivatives with 99% conversion at TTL/GBP4 ratio of 1:5 within 3 h, followed by fluorophore, biotin, and polyethylene glycol labeling via various bioorthogonal reactions such as SPAAC, Staudinger ligation and Staudinger-phosphite reaction. This did not affect the fluorescence and fluorescence-modifying properties of GFP or GFP-specific nanobodies. This study demonstrated the specificity of these modified nanobodies [56]. Considering the properties of nanobodies like better hydrophilicity, smaller molecular weight, higher stability, and lower immunogenicity in comparison to conventional monoclonal antibody shows the potency for applications in biochemistry and medicine. However, even though the Tub-tag labeled nanobodies introduce a fast and effective method, it is still needed to be further confirmed in vitro and in vivo, as nanobody has rapid renal clearance [57] and most nanobodies are still under clinical trials. To date, there is little evidence about the conjugation of tyrosine modified nanobodies with chemotherapeutic drugs, leaving a wide world for us to further explore. 

### 3.5. Trypsiligase and Subtiligase

N-terminal protein modification has appealed to biochemists to explore a new method for site-specific protein modification by taking advantage of only one N-terminus in each protein chain. Proteinase plays a vital role in enzymatic protein modification, whereas the hydrolysis property hampers its application scope. Hence, scientists have engineered enzymes to improve the defection and successfully synthesized some variants, such as trypsiligase [38] and subtiligase [58]. 

Trypsiligase is a fourfold trypsin variant K60E/N143H/E151H/D189K, derived from serine protease anionic rat trypsin II [59]. The esterase activity of variant D189K at S1 binding site for P1-tyrosine is low and the proteolytic activity is negligible. The K60E amino acid at the S1′ leads to specificity for P1′-arginine two orders of magnitude greater than that for other residues, and the histidine N143H and E151H at S2′ subsite shows 350-fold increased activity for P2′-histidine-containing substrates due to a unique recognition element with the presence of zinc ions [38,59]. All of these make trypsiligase obtain the ability to mediate N- or C-terminal protein modification on the basis of specific recognition sequence YRH (Figure 2E). POI equipped with N-terminal purification tag and YRH allows the trypsiligase to cleave the Y–R peptide bond, followed by bioconjugation reaction with the presence of acyl-4-guanidinophenyl ester (4-OGp) which bears artificial functionality. The C- terminal modification is a transpeptidation procedure, where an intermediate acyl-enzyme produced after cleavage reacts with Arg-His-containing acceptor peptide modified with desired functionality [38,59,60]. To confirm the site-specific modification potency of trypsiligase, Liebscher et al. introduced YRH and N-terminal Strep-tag II fusion to human Cyclophilin 18 (hCyp18). After being incubated with trypsiligase followed by fluorophore labeling, complete cleavage of Strep-tag II and labeling were observed [38]. Later, Liebscher and her co-workers developed this technology with click chemistry to produce homogeneous ADC. Following the procedure we described above, Fab anti-Her2 and Fab anti-TNFα derived from trastuzumab and certolizumab respectively were equipped with tetrazine through C-terminal modification during the enzymatic step, as modifying the N-terminal region of an antibody may affect its binding ability. For the chemical step, DM1 was conjugated to Fab-anti-Her2-YRH-Tet, producing Fab-anti-Her2-YRH-DM1 via inverse Diels-Alder click reaction, as well as PEG and carboxyfluorescein were conjugated to Fab-anti-TNFα-Tet [39]. 

Subtiligase is an engineered peptidase derived from serine protease subtilisin BPN’ from Bacillus amyloliquefaciens [58,61]. The two amino acid mutations enable subtiligase to catalyze a ligation reaction between an acyl donor peptide ester and the N-terminal α-amine of an acceptor peptide or protein. The change of catalytic serine to cysteine (S221C) contributed to the 1000–10,000-fold reduction in amidase activity, but only 3-fold reduction in esterase activity. The second mutation of proline to alanine (P225A) eased the steric crowding of active site and enhanced aminolysis efficiency. The S221C/P225A double mutant resulted in >10^7^ reduction in amidase activity compared to wildtype subtilisin, whereas there was a 10-fold increase in esterase activity compared to the S221C mutant [61]. As with other enzymes applied in the bioconjugation field, Weeks et al. employed subtiligase in enzymatic-step of site-specific protein modification, followed by click chemistry to introduce fluorophore, biotin, and cytotoxic drug (MMAE) into anti-GFP antibody, and confirmed barely unaffected affinity of conjugates after modification [62]. 

Trypsiligase and subtiligase are great potential tools in site-specific protein bioconjugation without the need for additional genetic manipulation. Nevertheless, the limitation of N-terminal protein modification remains that the N terminus may be inaccessible. Moreover, further research is needed to confirm the pharmacokinetics of these ADCs under complicated environments in cells or organisms. 

### 3.6. Phosphopantetheinyl Transferase

Phosphopantetheinyl transferases (PPTases), which catalyze the covalent binding of 4′-phosphopantetheinyl cofactor (Ppant) from coenzyme (CoA) to a highly conserved serine residue within acyl carrier proteins (ACPs) or peptidyl carrier proteins (PCPs), play an essential role in post-translational modification and have been exploited to site-specific protein modification owing to its broad substrate tolerance [40]. The most utilized PPTases are B. subtilis Sfp and E. coli AcpS, which allows for labelling of PCP and ACP sites specifically with Ppant [63,64]. As the relatively large size of PCP and ACP (80–120 residues) hampered their applicability, an 11 residue peptide termed ybbR tag with the sequence DSLEFIASKLA was discovered as an substrate for Sfp (Figure 2F) [65]. Later, two 12-residue peptide tags—S6 (GDSLSWLLRLLN) and A1 (GDSLDMLEWSLM)—wereidentified to site specifically label proteins by Sfp and AcpS, respectively. In addition, the orthogonality property of these two tags broadened their applicability in dual labeling applications [66]. 

PPTases can be used both in one step conjugation and two-step conjugation. In the field of antibody drug conjugation, Grunewald et al. inserted S6/ybbR into surface-exposed loops of the different constant domains of trastuzumab, and then CoA coupled with monomethyl auristatin F (MMAF) via maleimide chemistry was loaded to trastuzumab by Sfp. The conjugation in the CH1 domain showed optimal pharmacokinetics and least drug loss, while the CH2 domain conjugates suffered rapid clearance due to low thermal stability [41]. In line with the high efficiency of cell killing in vitro, tumor regression was induced by one ADC in a xenograft model [41]. Furthermore, this research group developed a two-step strategy, which conjugated CoA analogs coupled with bioorthogonal handles (ketone, azide, and alkyne groups) to antibodies followed by drug loading via bioorthogonal chemical reactions. The CoA analogs preparation was ATP-dependent and involved CoaA, CoaD, and CoaE, and these bioorthogonal CoA analogs were capable of using the short peptide tag with only six amino acids (DSLSWL) due to their structural similarity to natural PPTase substrate CoA-SH. Compared to the one-step approach, the two-step approach had higher conjugation yields, and HER2-positive cells were also killed at sub-nanomolar ADC concentrations. Moreover, the results showed that shorter linker length was prone to improve the conjugate stability in mice without affecting the cell killing potency of auristatin payloads and the suitable conjugation site for linker-payload attachment could also modulate extracellular stability of ADCs. Therefore, optimizing both factors allows ADCs to be highly stable in circulation, increasing the safety which can be a problem in clinical trials of ADCs [42]. 

Overall, PPTases provide an innovative way to produce homogeneous ADCs efficiently, and the dual labeling potency could be used to label hydrophilic functionalities or multiple drugs to ease the hydrophobicity and drug resistance issues.

### 3.7. Spyligase

Peptide tags are potent tools in site-specific protein modification owing to their minimal influence in protein function. Fierer et al. split a peptide tag termed SpyTag and a protein domain termed SpyCatcher from CnaB2 domain in the fibronectin adhesion protein FbaB of Streptococcus pyogenes. These two parts could form an isopeptide bond in minutes and enabled peptide–protein ligation (Figure 2G) [67]. The SpyCatcher/SpyTag protein ligase system was used for in vivo optical imaging. The result showed that the SpyTag/SpyCatcher fusions in the C-terminus did not affect the antigen affinity. The specific binding ability to targets and selective accumulation in xenografts suggested that SpyCatcher/SpyTag protein ligase system may be a potent methodology to produce ADCs, but immune response would occur due to addition of the protein SpyCatcher (15KD) [43]. Later, Fierer et al. split three modules termed SpyTag, KTag and SpyLigase, enabling peptide–peptide ligation. SpyTag and KTag are capable of forming an isopeptide bond irreversibly between Asp and Lys catalyzed by an opposed glutamate residue in SpyLigase [68]. Siegmund et al. dedicated the SpyLigase-mediated ligation to the engineering of site-specific antibody drug conjugates by fusing SpyTag to the C-terminus of anti-EGFR antibody cetuximab, and attaching MMAE to KTag via click chemistry. The results showed the specificity of the reaction, and also confirmed the high cytotoxicity in vitro with sub-nanomolar IC50 values. However, the conjugation required 15 equivalents of the toxic payload, and three equivalents of SpyLigase to SpyTag, which seemed cost consuming [44]. In addition, the ligation efficiency of the SpyLigase system was low in comparison to SpyTag/SpyCatcher system [43], and the highest DAR (1.76) of the tested ADCs was lower than the recommended range (2–4) [44]. In conclusion, extending this peptide tag ligation in site-specific protein labeling should be fruitful. Given the fact that both SpyCatcher/SpyTag protein ligase system and SpyLigase-mediated ligation have their merits and demerits, finding a compromise to improve the shortcomings should be taken into consideration. 

### 3.8. O6-Alkylguanine-DNA Alkyltransferase (AGT) (SNAP-Tag)

This method, which is also called the SNAP-tag method, is based on the predominant pathway of DNA repair, which involves the transfer of the alkyl group from the DNA to a cysteine acceptor site with O6-alkylguanine-DNA alkyltransferase (AGT) enzyme [69]. The gene encoding of AGT was cloned first by Sedgwick from wild *E. coli* [70] and later by Tano and his collogues who completed the cDNA clone encoding for the human O6-alkylguanine [71].

In this pathway, the acceptor cysteine cannot be regenerated and the number of O6-alkylguanines that can be repaired is equal to the number of active alkyltransferase molecules. AGT can induce the repair of O6-alkylguanine in double-stranded DNA via transferring the alkyl group from the DNA to an internal cysteine residue in the AGT protein irreversibly (suicide reaction), forming a thioether (S-alkylcysteine) and thus the AGT protein deactivates [72,73]. According to this property, Johnsson et al. described a general method for the covalent labeling of hAGT fusion proteins with O6-benzylguanine (BG) specifically. Here a 20kDa engineered version of hAGT (SNAP-tag) with higher reactivity towards BG modified molecules has been generated. The cysteine residue of SNAP-tag protein reacts with the benzyl group of the O6-benzylguanine through a nucleophilic substitution, providing a 1:1 stoichiometry between recombinant SNAP-tag fusion protein and BG-modified molecule (Figure 2H) [45]. 

The SNAP-tag has been applied to label single recombinant proteins with BG-modified fluorophores in complex protein mixtures and living cells. Furthermore, the SNAP-tag based conjugation method has been used for high-resolution imaging, tracking single-molecules, determining protein–protein interactions, tagging plasma membrane proteins, determining protein half-life and studying the functions of different proteins [74,75,76,77,78,79].

As an additional advantage of the technology, different BG-modified effector molecules, such as photosensitizers, toxins, or fluorophores, can be conjugated to SNAP-tag in a site-specific manner without affecting the structure and function of the recombinant ligand [46,80,81]. Therefore, SNAP-tag can overcome the limitation of heterogeneous products in ADCs, especially the affinity constant of a single chain fragment variable (scFv) in a fusion protein. The scFv antibody derivatives are one of the most popular recombinant antibodies. They are considerably smaller (26–27 kDa) [82] but retain a complete structure of the binding region of antibodies. The scFv is composed of VH and VL domains that are joined by a flexible peptide linker. This linker consist generally of a 15 amino acid residue (Gly4Ser)3 linker [83]. 

Over the last several years, the intrinsically monovalent and highly specific conjugation activities of SNAP-tag have been exploited to equip several recombinant antibody fragments targeting different cancer cell-surface receptors with effector molecules (ref.).

A BG-modified auristatin F (AURIF) has been coupled with EGFR and Her2 specific recombinant antibody fragments SNAP-tag proteins. Conjugating the AURIF to anti-Her2 and anti-EGFR recombinant antibodies significantly improved the specificity of AURIF and increased its cytotoxicity by more than 30-fold toward Her2 expressing breast cancer cells [46]. 

Furthermore, the highly potent near-infrared (NIR) imaging/photosensitizer IRDye700 (phototheranostics) agent was conjugated to several recombinant antibody fragments targeting different cancer cell-surface receptors using SNAP-tag technology to generate a panel of NIR photoimmunotheranostics (NIR-PIT) agents [47,81,84,85,86]. 

Each of the components of our NIR-PIT approach had minimal toxicity when presented alone. Thus, toxicity was achieved only when the components were applied together at the appropriate place and time. In addition, a significant correlation was found between cytotoxicity and cell surface receptor density [81]. These findings indicate that our NIR-PIT agents have a high therapeutic potential and offer a failsafe system to prevent off-target effects.

However, the 1:1 stoichiometry site-directed labeling properties of SNAP-tag could be seen as a limitation for those cases where an increasing the DAR is required. As a solution, generating BG-modified payload bearing multiple cytotoxic molecules could be used to overcome this limitation. 

## 4. Conclusions and Perspectives

Over the last several years, an increased number of enzyme-based site-specific conjugation methods have been developed to generate antibody conjugates with nearly defined DAR, which represent unified pharmacokinetic, safety, and efficacy properties. Most of these methods are based on genetic incorporation of short peptides or small proteins, which provide unique conjugation sites that allows specific coupling reactions (Table 1). 

Furthermore, Different enzyme-based conjugation methods can be combined with chemical conjugation methods e.g., cysteine conjugation methods for dual labeling an individual antibody with different effective molecules [87,88].

Although these methods have been applied successfully to arm antibodies with effector molecules, some of them still need high correspondence enzyme concentration and high equivalents of effector molecules and generally show lower flexibility in case higher DAR is required. 

Furthermore, these short peptides and proteins are generally prokaryotic derivatives or genetically modified human derivatives, which could induce immune response, e.g., neutralizing immune responses, which is crucial for both safety and efficacy for correspondence antibody conjugates used in clinical applications. Therefore, intensive immunogenicity assessments for generated antibody conjugates are required during product development.

## Figures and Tables

**Table 1:** List of enzyme-based site-specific conjugation methods including their substrate, coupling method and their applications.

Conjugation Method	Substrate	Coupling Method	Applications	References
Sortases	LPXTG	Labeling peptide	Full length antibody, Fab	[32]
Formylglycine-based methods	LCTPSR	Aldehyde coupling chemistry	Full length antibody	[34,35]
Transglutaminases	LLQGA	Labeled alkyl- or oligo-amine	Full length antibody	[36]
Tubulin tyrosine ligase	VDSVEGEGEEEGEE, Tub-tag	Labeled tyrosine	scFv	[37]
Trypsiligase and subtiligase	YRH	Labeled peptide	Fab	[38,39]
Phosphopantetheinyl transferase	DSLEFIASKLA	Labeled CoaA	Full length antibody	[40,41,42]
Spyligase	AHIVMVDAYKPTK	Peptide-peptide ligation	Full length antibody	[43,44]
SNAP-tag	BG-modified molecules	Irreversible transfer of an alkyl group to a cysteine residue	scFv	[45,46,47]
